# Patients with anorexia nervosa perceive higher emotional connectedness to their parents than bulimia nervosa patients independent of objective family factors

**DOI:** 10.1007/s40211-025-00558-y

**Published:** 2025-11-26

**Authors:** Elias Fischer, Noah Lensch, Günter Reich, Thomas Meyer

**Affiliations:** Department of Psychosomatic Medicine and Psychotherapy, University of Göttingen, Von-Siebold-Str. 5, Göttingen, Germany

**Keywords:** Eating disorders, Family perception, Emotional connectedness, Individual autonomy, Subjective Family Image Test, Essstörungen, Familienwahrnehmung, Emotionale Bindung, Individuelle Autonomie, Subjectives Familienbild

## Abstract

**Background:**

Psychological constructs of individual autonomy (IA) and emotional connectedness (EC) differ between patients with anorexia nervosa (AN) and bulimia nervosa (BN); however, it is unclear whether objective family factors modulate these associations.

**Methods:**

In a cohort of 445 participants from the FamFINED study (FAMily Factors INvolved in Eating Disorders) with eating disorders (EDs) diagnosed as AN (*n* = 232) or BN (*n* = 213), we assessed objective psychosocial family factors as well as the IA and EC constructs using the self-rated Subjective Family Image Test (SFIT) questionnaire.

**Results:**

While the two ED entities did not differ with respect to IA in univariate analysis (*p* = 0.355), patients diagnosed with AN had higher perceived EC than participants with BN, as assessed by a lower score for the difference between the actual and desired family image (16.0 ± 15.8 vs. 21.6 ± 17.6, *p* < 0.001). In a regression model adjusted for age, body-mass index, living in parental home, and parental separation, we found that the EC score used as the dependent variable differed significantly between the two ED entities (expβ = 4.8, 95% confidence interval = 0.54–9.11, *p* = 0.028). In contrast, no association was observed between IA and ED diagnoses using the same set of confounders (*p* = 0.717).

**Conclusion:**

The higher perceived EC in AN patients compared to those diagnosed with BN indicates more intense feelings of family solidarity, regardless of objective family factors.

## Abstract (de)

**Hintergrund:**

Die psychologischen Konstrukte der individuellen Autonomie (IA) und der emotionalen Verbundenheit (EC) unterscheiden sich zwischen Patientinnen mit Anorexia nervosa (AN) und Bulimia nervosa (BN); es ist jedoch unklar, ob objektive familiäre Faktoren diese Zusammenhänge beeinflussen.

**Methoden:**

In einer Kohorte von 445 Teilnehmerinnen aus der FamFINED-Studie (Family Factors Involved in Eating Disorders) mit Essstörungen (ED), die als AN (*n* = 232) oder BN (*n* = 213) diagnostiziert wurden, bewerteten wir objektive psychosoziale Familienfaktoren sowie die Konstrukte IA und EC anhand des selbstausgefüllten Fragebogens Subjectives Familienbild (SFB).

**Ergebnisse:**

Während sich die beiden ED-Entitäten in der univariaten Analyse hinsichtlich IA nicht unterschieden (*p* = 0,355), wiesen Patientinnen mit AN eine höhere wahrgenommene EC auf als Teilnehmerinnen mit BN, was anhand eines niedrigeren Werts für die Differenz zwischen dem tatsächlichen und dem gewünschten Familienbild bewertet wurde (16,0 ± 15,8 vs. 21,6 ± 17,6; *p* < 0,001). In einem Regressionsmodell, das hinsichtlich Alter, Body-Mass-Index, Wohnen im Elternhaus und Trennung der Eltern adjustiert wurde, stellten wir fest, dass sich der als abhängige Variable verwendete EC-Wert zwischen den beiden ED-Entitäten signifikant unterschied (expβ = 4,8; 95% Konfidenzintervall 0,54–9,11; *p* = 0,028). Im Gegensatz dazu wurde unter Verwendung derselben Störfaktoren kein Zusammenhang zwischen IA und der ED-Diagnose beobachtet (*p* = 0,717).

**Schlussfolgerung:**

Die im Vergleich zu BN-Patienten höher wahrgenommene EC bei AN-Patienten deutet auf ein stärkeres Gefühl der familiären Solidarität hin, unabhängig von objektiven Familienfaktoren.

## Introduction

Anorexia nervosa (AN), characterized by restrictive food intake generally resulting in a significantly reduced body-mass index (BMI), and bulimia nervosa (BN), defined by recurrent episodes of binge eating typically followed by compensatory behaviors, are increasingly conceptualized as neurodevelopmental disorders [1, 2]. The onset of these eating disorders (EDs) during peripuberty often coincides with periods of critical developmental transitions, which may exert profound effects on family dynamics, particularly within the parent-child triad. Psychological changes occurring during this vulnerable developmental stage frequently include the emergence of mood disturbances, body dissatisfaction, and concerns regarding body shape and weight, all of which may serve as precipitating factors for the development of AN and BN [3]. The ability to establish meaningful interpersonal relationships arises from the changing interplay between individual autonomy (IA) and emotional connectedness (EC), which plays a central role in identity development during adolescence [4].

A reliable tool for the assessment of the patients’ perceived IA and EC is the established Subjective Family Image Test (SFIT) [5, 6]. Employing the SFIT questionnaire, a retrospective, observational study in 332 participants with EDs found that patients with BN reported significantly lower levels for the psychological construct of EC than those with AN, relative to their healthy sisters [7]. Karwautz et al. (2002) reported that individuals with BN demonstrated lower scores for both individual autonomy (IA) and emotional connectedness (EC) compared to those with AN, when both groups were considered in relation to healthy controls [8]. In a subsequent study, the same research group observed that patients with AN exhibited significantly reduced IA relative to their healthy sisters; however, no significant differences were identified between these groups with respect to EC scores [9].

Given that mental and environmental factors change over the course of adolescence, we wondered whether the reported difference between the two ED entities with respect to the constructs IA and EC could still be observed, when objective family measures are taken into account by means of adjusted models. To this end, we tested the hypothesis that the associations between the ED diagnoses and the psychological constructs of subjective family functioning remain stable irrespective of important objective family factors changing over time.

## Methods

### Study design and participants

All participants from the FamFINED study (FAMily Factors INvolved in Eating Disorders) included in the present analysis were diagnosed with ED according to the 10^th^ International Statistical Classification of Diseases and Related Health Problems (ICD-10) or its predecessor as reported by the World Health Organization (WHO). The diagnoses included BN (ICD-10 codes F50.2 and F50.3) and AN (ICD-10 codes F50.0 and F50.1). All patients were treated from 1991 to 2017 at the Department of Psychosomatic Medicine and Psychotherapy, University Medical Center Göttingen [10, 11]. The participants were predominantly in adolescence or early adulthood, which reflects the typical age distribution of EDs [12]. Specialists in psychosomatic medicine and psychotherapy or licensed clinical psychologists conducted a comprehensive and systematic diagnostic assessment using interviews and clinical tests to confirm the diagnosis. As an integral part of the study, patients were asked to complete a set of self-report questionnaires, which collected a range of demographic and clinical information, including age, gender, difference between actual and desired weight, and parental education levels. Additional clinical and psychosocial data were gathered including body-mass index (BMI), defined as kilograms per square meter of body surface area, as well as the current living situation (e.g., living alone or with parents), parental marital versus separation status, and presence of siblings. Psychological distress was assessed using the Global Severity Index of the SCL 90R (GSI) [13]. The survey was registered in the ClinicalTrials.gov register (#NCT05339165), and participation of eligible patients was voluntary. Written informed consent was obtained from participants of legal age and/or legally authorized representatives. The study adhered to the ethical principles outlined in the 1964 Declaration of Helsinki and received ethical approval from the Institutional Ethical Review Board of the University of Göttingen Medical Center under the number #32/1/17.

### Subjective family image test

The SFIT questionnaire on subjective family perception was used to assess the discrepancy between the participants’ perceived actual and ideal family environment. The questionnaire designed by Mattejat and Scholz (1994), which is a self-report instrument completed by the study participants, measures subjective perceptions of interactions within the father-mother-child triad [5]. The SFIT was developed for use in adolescents aged 12 years or older as well as adults. The concept of subjective family perception is based on two central dimensions with valence representing the degree of EC and potency as a measure of IA [6]. On a 7-point Likert scale (−3 to +3), six pairs of opposing adjectives are assessed. The IA scale is based on three bipolar adjective dimensions reflecting opposing interpersonal traits, such as confident vs. anxious, independent vs. dependent, and decisive vs. indecisive. The construction of the EC scale is based on the following set of contrasting adjectives: warm-hearted vs. cool, understanding vs. intolerant, and interested vs. disinterested. A total family sum score was calculated for each of the two dimensions by aggregating the associated numerical values marked on the questionnaire. The evaluation involves calculating family sum scores for valence and potency separately for both the actual and the desired family perceptions. This allows for the computation of discrepancy scores, reflecting the extent to which the perceived family environment deviates from the individual’s ideal. These values can then be interpreted such that greater differences in the valence scores indicate a lower perceived level of EC, whereas smaller differences suggest a higher perceived level of EC. The SFIT demonstrates satisfactory reliability and validity with an average Cronbach’s α of 0.61 for the IA scale and 0.81 for the EC scale [7]. In our study population, we confirm the high internal consistency of the SFIT instrument with a Cronbach’s α of 0.88 for the IA scale and 0.92 for the EC scale. Parallel test reliability ranged from *r* = 0.61 to *r* = 0.80. Test-retest reliability values ranged from *r*_*tt*_ = 0.66 to *r*_*tt*_ = 0.82 after a 2-week interval, and from *r*_*tt*_ = 0.55 to *r*_*tt*_ = 0.69 after 11 months [7].

### Data analysis

The data for this retrospective, observational study were obtained through electronic transfer from the paper-based questionnaires completed by the participants. Statistical analyses were performed by using the Statistical Package for the Social Sciences, version 30 (SPSS, IBM, Armonk, NY, USA). Categorical variables were presented as percentages and continuous variables as means with standard deviations. In the absence of a control group without an ED diagnosis, a median split approach was adopted to enable comparative analysis. Participants in the study were stratified according to whether their difference in the family sum valence was above or below the median, and the two groups were compared by using the Student’s *t*-test. Normal distribution of data was routinely checked. Levene’s test was applied to evaluate homogeneity of variances between the two groups. The χ^2^ test was used for categorical variables such as living situation, presence of siblings or parental education in order to detect differences between the groups above and below the median. Linear associations were analyzed using Pearson’s correlation between continuous variables (including patient’s age, maternal and paternal age, BMI, GSI, and the difference to desired weight) and the difference in the family sum valence. Finally, a multivariate regression model was computed to study whether the discrepancy between the desired and perceived family image differed between the two ED entities, when the following confounders were taken into consideration: age of the patient, BMI, living with parents, and parental separation. The relative influence of predictors was expressed by using the regression coefficient β. For each variable, the 95% confidence interval (95%CI) was calculated. A significance level of *p* < 0.05 was applied. Due to the exploratory nature of this investigation, no adjustments for multiple testing were performed.

## Results

### Characterization of the study cohort

The mean age in the total study population was 21.7 ± 6.2 years. Patients diagnosed with AN were significantly younger than BN patients (20.9 ± 6.3 vs. 22.5 ± 6.1 years, *p *= 0.006). They had a lower BMI (16.8 ± 1.7 vs. 20.4 ± 2.7 kg/m², *p *< 0.001) and GSI (0.97 ± 0.60 vs. 1.26 ± 0.64, *p *< 0.001), and lived more frequently in their parental homes (27.8 vs. 10.6%, *p *< 0.001). Compared to AN patients, study participants diagnosed with BN had a significantly lower EC, defined as a higher difference between the actual and desired family perception (21.6 ± 17.6 vs. 16.0 ± 15.8, *p* < 0.001). The study cohort (*n* = 445) was divided into two groups based on the median of the difference in the family sum valence, dichotomizing the patients into those with low and high perceived EC. Patients with sum scores below the median, indicating higher perceived EC, were significantly younger than those with a lower EC (20.96 ± 5.97 years vs. 22.43 ± 6.41, *p* = 0.013). They also had a significantly lower BMI compared to those above the median (18.2 ± 2.8 kg/m^2^ vs. 18.8 ± 3.0, *p* = 0.031). Furthermore, their level of psychological distress, as measured by the GSI, was significantly lower than in those with reduced EC (1.00 ± 0.64 vs. 1.23 ± 0.62, *p* < 0.001). These patients also exhibited a smaller difference between their current and desired weight (0.76 ± 5.81 kg vs. 1.97 ± 6.09, *p* = 0.045). Additionally, a significantly smaller proportion of patients with lower EC still lived with their parents compared to those with higher EC values (15.3% vs. 23.6, *p* = 0.031). Participants in the group above the median reported a significantly higher IA compared to the group below the median (18.9 ± 15.9 vs. 7.4 ± 14.4, *p* < 0.001). A significantly greater proportion of patients with scores above the median reported that their parents lived separately (18.3% vs. 11.4, *p* = 0.043) and were significantly more likely to be diagnosed with BN (57.2% vs. 39.1, *p* < 0.001) compared to those with a higher EC. In contrast, study participants below the median were more frequently diagnosed with AN (60.9% vs. 42.8, *p* < 0.001). No significant differences were found between the groups regarding parental age, sex, or educational background. Clinical and epidemiological data are presented in Table 1.

**Table 1:** Characterization of the total study population and comparison of the difference in the family sum valence along the median split

	Total study cohort (*n* = 445)	Below median (*n* = 230)	Above median (*n* = 215)	*P* value
Age patient (years)	22.05 ± 6.57	20.96 ± 5.97	22.43 ± 6.41	**0.013**
Age mother	50.85 ± 7.59	49.92 ± 7.15	50.83 ± 7.19	0.186
Age father	53.55 ± 7.85	52.69 ± 7.51	54.04 ± 7.72	0.066
Sex (female, %)	97.3	97.8	96.7	0.480
Body-mass index (kg/m^2^)	18.6 ± 3.0	18.2 ± 2.8	18.8 ± 3.0	**0.031**
Global Severity Index	1.11 ± 0.64	1.00 ± 0.64	1.23 ± 0.62	**<** **0.001**
Difference to desired weight (kg)	1.47 ± 6.3	0.76 ± 5.81	1.97 ± 6.09	**0.045**
Education level mother (high school degree, %)	36.9	38.1	35.6	0.593
Education level father (high school degree, %)	48.8	53.1	44.2	0.064
Education level patient (high school degree, %)	51.5	51.3	51.6	0.946
Living alone (%)	45.4	44.0	46.9	0.546
Living in parental home (%)	19.6	23.6	15.3	**0.031**
Parents separated (%)	14.7	11.4	18.3	**0.043**
Sibling (%)	83.7	83.8	83.6	0.971
Individual autonomy	12.9 ± 16.2	7.4 ± 14.4	18.9 ± 15.9	**<** **0.001**
Diagnosis of AN (%)	52.1	60.9	42.8	**<** **0.001**
Diagnosis of BN (%)	47.9	39.1	57.2	**<** **0.001**

*AN* anorexia nervosa, *BN* bulimia nervosa

### Correlations between the difference in the family sum valence and family factors

**Fig. 1:**
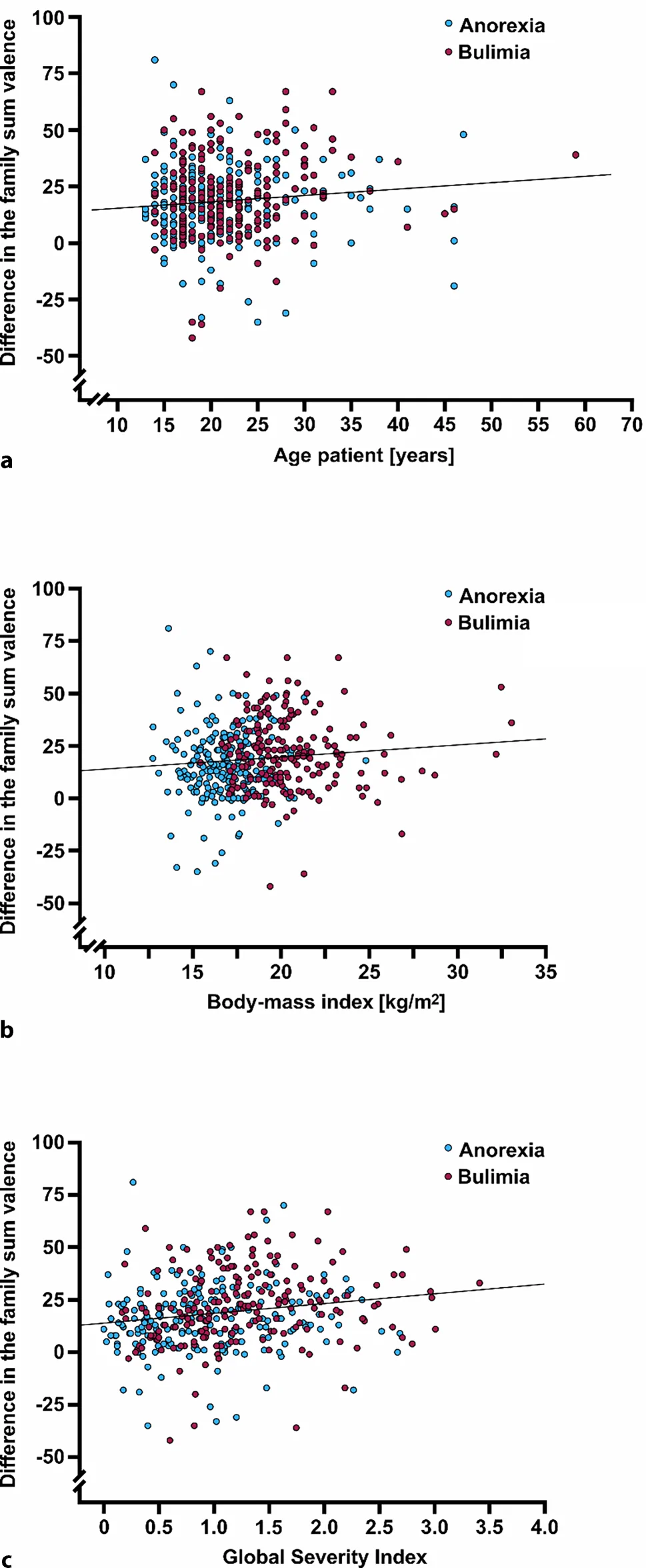
Relationships between the difference in the family sum valence and the age of the patient (**a**), body-mass index (**b**), and Global Severity Index (**c**)

Using Pearson’s correlation, significant associations were observed between patients’ perceived EC and several family factors. In the AN group (*n* = 232), significant correlations were found between the difference in the family sum valence and the age of the father (r = 0.136, *p* = 0.042), as well as the difference to desired weight (r = 0.153, *p* = 0.033). No other variables showed statistically significant associations in AN patients. In the BN group (*n* = 213), significant positive correlations were identified between the difference in the family sum valence and both the patient’s age (r = 0.147, *p* = 0.032) and the GSI (r = 0.183, *p* = 0.009). All other variables showed no significant associations with perceived EC in BN patients. Regarding the total study cohort (*n* = 445), positive correlations were found between the difference in family sum valence and the age of the patient (r = 0.104, *p* = 0.028, Fig. 1a), the mother’s (r = 0.096, *p* = 0.044) and the father’s age (r = 0.109, *p* = 0.023), BMI (r = 0.099, *p* = 0.042, Fig. 1b), GSI (r = 0.173, *p* < 0.001, Fig. 1c), and the difference between current and desired weight (r = 0.150, *p* = 0.003). These correlations are illustrated in Table 2.

**Table 2:** Correlations between the difference in the family sum valence and important family factors

	Total study cohort (*n* = 445)		Anorexia nervosa (*n* = 232)		Bulimia nervosa (*n* = 213)	
	PCC	*P* value	PCC	*P* value	PCC	*P* value
Age patient (years)	0.104	**0.028**	0.022	0.735	0.147	**0.032**
Age mother (years)	0.096	**0.044**	0.100	0.134	0.080	0.247
Age father (years)	0.109	**0.023**	0.136	**0.042**	0.066	0.338
Body-mass index (kg/m^2^)	0.099	**0.042**	0.014	0.842	−0.029	0.681
Global Severity Index	0.173	**<** **0.001**	0.090	0.195	0.183	**0.009**
Difference to desired weight	0.150	**0.003**	0.153	**0.033**	−0.030	0.678

*PCC* Pearson’s correlation coefficient

### Emotional connectedness significantly differed between the two ED entities

In a linear regression model, we investigated the relationship between the difference in the family sum valence used as independent variable and the diagnosed ED entity (AN or BN; Table 3). Potential confounding variables included in the model were age of the patient (in years), BMI (kg/m^2^) and, as family factors, parents separated (%), and living in parental home (%). Our data in the adjusted model confirmed the significantly higher EC in patients diagnosed with AN as compared with those with BN (β = 4.823, 95%CI = 0.535–9.111, *p* = 0.028; Table 3). The confounding variables and family factors did not reach statistical significance.

**Table 3:** Results from a linear regression model with the difference in the family sum valence as the dependent variable and the eating disorder (ED) diagnosis as the independent variable adjusted to the indicated variables

Model: (R^2^ = 0.036, *p* = 0.013)	Regression coefficient β	95% confidence interval	*P* value
Age patient (years)	0.161	[−0.136; 0.458]	0.287
Body-mass index (kg/m^2^)	−0.093	[−0.836; 0.650]	0.806
Parents separated (%)	2.457	[−2.158; 7.072]	0.296
Living in parental home (%)	−2.993	[−7.466; 1.479]	0.189
ED (anorexia vs. bulimia)	4.823	[0.535; 9.111]	**0.028**

## Discussion

The results of the presented study from a large cohort of 445 participants diagnosed with EDs confirm that, in univariate analysis, patients with AN perceive higher EC than BN patients, indicating that they feel more emotionally attached to their family. In an adjusted linear regression model, the higher perceived EC in AN patients as compared to BN patients remained stable. These data show that, independent of age-related family factors such as separated parents and living in parental home, the perceived EC differs significantly between the diagnoses of AN and BN. Additionally, in the total study cohort, significant positive correlations were found between the difference in the family sum valence and the following variables: patient’s age, maternal and paternal age, BMI, GSI, and the difference between actual and desired weight.

These findings are consistent with previous research indicating that different subtypes of EDs are associated with varying degrees of the perceived EC [7–9, 14–16]. Fornari et al. (1999) reported that, in terms of family functioning, the diagnosis of BN was a stronger predictor for impaired family functioning than allocation to the AN group [14]. Similarly, von Boetticher et al. (2014) found a higher degree of EC towards the family among patients with AN compared to those with BN [16]. Further results indicate that strong family connectedness serves as a protective factor, whereas low levels of connectedness increase the risk for the onset and persistence of AN [17]. Furthermore, Fäldt Ciccolo (2008) suggested that individuals with AN may underreport family dysfunction due to their heightened need for emotional control, as well as tendencies toward idealization and denial regarding their current family circumstances [18]. Additionally, individuals with AN often describe remarkably positive family relationships, frequently characterizing them as harmonious and marked by emotional closeness and fulfillment [15, 19, 20].

One possible explanation for this positive view of family circumstances could be a link to alexithymia, describing the dysfunction in the perception and interpretation of internal emotional experiences, particularly in patients with AN [21, 22]. In this context, Fox (2009) proposed that AN may originate from a fundamental impairment in recognizing and expressing one’s own emotional states [23]. This conceptualization aligns with current findings suggesting that alexithymic traits play a particularly prominent role in the emotional dysregulation observed among individuals with AN, pointing to the possibility that deficits in emotional awareness and expression may contribute to the maintenance of restrictive eating behaviors [21]. In contrast, young women diagnosed with BN perceive their parents as emotionally fragile and limited in self-care, which hinders the formation of their own autonomy and emotional processing, potentially leading to developmental impairments across psychological, somatic, and relational domains [24, 25]. Previous research has indicated that individuals with BN perceive their families as exhibiting lower cohesion compared to those with AN or healthy controls [26].

Several studies have further suggested that a lower age gap between the patient and their parents is associated with a higher symptom severity [27–30]. In our study population, the BMI showed a significant positive correlation with the difference in family sum valence. A linear regression model demonstrated that the family sum valence was associated with the ED diagnosis. With regard to the observed positive correlations between lower perceived EC and both the GSI and the difference to desired weight, the current literature does not provide any established explanations or supporting evidence. Therefore, further research is needed to verify or refute these findings.

This study has several limitations that should be acknowledged. The information on self-perception of the family environment was solely based on self-reports of the patients using the established and well-validated SFIT questionnaire which ensured anonymity and discretion. Additionally, the study did not involve randomization of participants. Furthermore, no comparison was made with a healthy control group as only patients diagnosed with EDs were included. Given the observational and retrospective nature of the survey, the study design does not allow any causal conclusions. No information can be provided regarding different subgroups of the two diagnoses, such as the bulimic and restrictive type of AN or atypical BN. The influence of various categorical and continuous variables on the ED in patients diagnosed with AN or BN should be further explored in future studies. However, the study also has advantages as it examines a well-characterized study population. The patients were examined and diagnosed by professionally trained specialists in the field of psychosomatics. Moreover, the large number of participants included in the study deserves special mention.

In summary, our study demonstrates that patients with anorexia nervosa perceive higher emotional connectedness (EC) to their family members than bulimia nervosa patients independent of objective family factors, such as separated parents or living in a parental home. Further studies are needed to better understand the relationships between the perception of EC and objective family factors.

## Data Availability

The datasets generated during the study are available from the corresponding author on reasonable request.
